# Translating nanoEHS data using EPA NaKnowBase and the resource description framework

**DOI:** 10.12688/f1000research.141056.1

**Published:** 2024-03-08

**Authors:** Holly M. Mortensen, Bradley Beach, Weston Slaughter, Jonathan Senn, Antony Williams, William Boyes

**Affiliations:** 1 ORD-CPHEA, US Environmental Protection agency, Research Triangle Park, NC, 27711, USA; 2 Appointee at the Office of Research and Development, US Environmental Protection Agency, Oak Ridge Institute for Science and Education (ORISE), Research Triangle Park, NC, 27711, USA; 3 Department of Biology, Duke University, Durham, NC, USA; 4 Oak Ridge Associated Universities, Oak Ridge, Tennessee, USA; 5 Metabolon, Inc. Precision Metabolomics, Durham, NC, USA; 6 ORD-CCTE, US Environmental Protection Agency, RTP, NC, 27711, USA

**Keywords:** nanomaterial; database; ontology

## Abstract

**Background:**

The U.S. Federal Government has supported the generation of extensive amounts of nanomaterials and related nano Environmental Health and Safety (nanoEHS) data, there is a need to make these data available to stakeholders. With recent efforts, a need for improved interoperability, translation, and sustainability of Federal nanoEHS data in the United States has been realized. The NaKnowBase (NKB) is a relational database containing experimental results generated by the EPA Office of Research and Development (ORD) regarding the actions of engineered nanomaterials on environmental and biological systems. Through the interaction of the National Nanotechnology Initiative’s Nanotechnology Environmental Health Implications (NEHI) Working Group, and the Database and Informatics Interest Group (DIIG), a U.S. Federal nanoEHS Consortium has been formed.

**Methods:**

The primary goal of this consortium is to establish a “common language” for nanoEHS data that aligns with FAIR data standards. A second goal is to overcome nomenclature issues inherent to nanomaterials data, ultimately allowing data sharing and interoperability across the diverse U.S. Federal nanoEHS data compendium, but also in keeping a level of consistency that will allow interoperability with U.S. and European partners. The most recent version of the EPA NaKnowBase (NKB) has been implemented for semantic integration. Computational code has been developed to use each NKB record as input, modify and filter table data, and subsequently output each modified record to a Research Description Framework (RDF). To improve the accuracy and efficiency of this process the EPA has created the OntoSearcher tool. This tool partially automates the ontology mapping process, thereby reducing onerous manual curation.

**Conclusions:**

Here we describe the efforts of the US EPA in promoting FAIR data standards for Federal nanoEHS data through semantic integration, as well as in the development of NAMs (computational tools) to facilitate these improvements for nanoEHS data at the Federal partner level.

## Highlights

• This work focuses on methods to improve interoperability of nanoEHS data.
• This work promotes FAIR data standards for nanoEHS data for the US Federal Government.
• Through the use of the NAMs presented here, we are able to translate nanoEHS data produced by US Federal Agency partners.
• The methods presented here facilitate traversing the separate and diverse nanoEHS data to address human health and environmental concerns.

**Abbreviations**
API: Application Programming Interface
ATSDR: Agency for Toxic Substance and Disease Registry
CCD: The EPA CompTox Chemical Dashboard
CPSC: Consumer Product Safety Commission
DIIG: Database Interoperability Interest Group (subgroup under NNI-NEHI)
EHLC: NIEHS Environmental Health Language Collaborative (EHLC)
EHS: Environmental health and safety
ENM: Engineered nanomaterials
EPA: Environmental Protection Agency
EU: European Union
FDA: Federal Drug Administration
FAIR: Findable, Accessible, Interoperable, Reusable
nanoEHS: nano Environmental Health and Safety
NAMs: New Approach Methodologies
NEHI: Nanomaterial Environmental Health Implications workgroup (under the NNI)
NIOSH: National Institute for Occupational Safety and Health
NKB: NaKnowBase
NLP: Natural Language Processing
NNI: National Nanotechnology Initiative
ORD: Office of Research and Developement
OSHA: Occupational Safety and Health Administration
RDF: Research Description Framework
SQL: Structured Query Language
US: United States

## Introduction

Engineered nanomaterials (ENM), usually defined as purpose-built materials with at least one dimension between approximately 1-100 nm, are a central component of the nanotechnology revolution and are increasingly used in a wide variety of industrial and commercial applications. The diverse applications of ENM include such widespread sectors as industrial catalysts and intermediates, electronics, health care, automotive materials, food containers, drugs, personal care products such as toothpaste and skin creams, sporting equipment, nano-enabled pesticides, and many more. The overall market size for nanotechnology is estimated to be $1.76 billion in 2020 and projected to exceed $33 billion by 2030 ( Allied Market Research 2021).

Responsible development of nanotechnology must include consideration of the potential implications for environmental health and safety (EHS). The widespread manufacture and application of ENM raises the potential for release of ENM at various points along the product life cycles ranging from initial synthesis and manufacture to product use, end-of-life and disposal. The release of ENM brings possible exposures to environmental species or humans ( Boyes, Thornton et al. 2017). The US federal government includes numerous agencies responsible for assuring the safety of the public and/or the natural environment, according to applicable statutes, including any potential risks associated with applications of ENM. For example, federal agencies may consider potential exposures in the workplace (NIOSH and OSHA), via foods, drugs and cosmetics (FDA), consumer products (CPSC), in hazardous waste sites (ATSDR), and via air, water, pesticides or as regulated toxic substances (EPA). The efforts of federal agencies to support the advancement of nanotechnology is coordinated through the National Nanotechnology Initiative (NNI, https://www.nano.gov/), and the evaluation of Environmental Health and Safety (EHS), coordinated through the Subcommittee on Nanoscale Science, Engineering, and Technology, and the Nanomaterial Environmental Health Implications (NEHI) workgroup under the NNI.

The various agencies responsible for ENM EHS have complementary interests in assessing the potential hazards of ENM. Uniformly, these assessments are challenged by limitations of data regarding the actions of ENM across their life cycle. This is true despite a large and growing scientific literature on nanotoxicology. There is a need for coordination and integration of nano EHS data to facilitate safety and risk assessments, and to further nanomaterial science through “big-data” assessments such as meta-analyses and quantitative structure-activity relationships. Nanoinformatics, the compilation, integration, and assessment of nanoEHS data, is an important component of nanoEHS, and has been the focus of concerted international efforts ( Haase 2018). The US-EU roadmap outlines a vision for data standards and organization that allows for computational re-use; however, all but a single contributed dataset at the time of writing is generated by EU initiatives.

Nanoinformatics efforts in the US have primarily been agency specific, whereby datasets are not interoperable and most often not accessible outside of any given agency. Efforts to coordinate data have been hindered by diversity of information, and format incongruities. The NEHI Database and Informatics Interest Group (DIIG) has recently formed a federal consortium to establish a standard protocol for the mapping of controlled vocabularies that is both consistent amongst the US Federal partner datasets, and between US and International efforts [NNI 2011 Research Strategy Update, 2023; Conference Proceedings, 2023]. The US EPA has contributed computational tools and training materials to further this effort.

The US EPA developed NaKnowBase (NKB), a database containing nanoEHS results published in peer-reviewed journals by EPA’s Office of Research and Development (ORD) ( Boyes, Beach et al. 2022). The NKB is currently implemented as a relational database using the structured query language (SQL). Though SQL is a universal language, access to the data included in a database requires some knowledge of the language, and potentially further manipulation of the data that may be beyond the capabilities of some potential users. Short of creating a graphical user interface (GUI) and hosting individual datasets externally, which is not a cost- effective solution for any one dataset, it has become important to provide NKB information in a more accessible format. One way this has been accomplished by the US EPA is by providing NKB data to EPA’s CompTox Chemicals Dashboard (CCD) [ Williams et al. 2017; Lowe and Williams, 2021], whereby NKB nanomaterials are linked to multiple points of information related to chemical toxicity.

The process of coordinating EPA NKB data with data from other US federal agencies, or other external databases, and CCD, requires consistency in nomenclature, and the ability to translate diverse terminology to common terms. This “Common Language” for nanoEHS parallels similar current efforts in the health sciences ( NIEHS 2023) and, to our knowledge is the first effort in the “FAIRification” of nanoEHS data. The goals and expected outcome of this work and subsequent efforts are to enable improved assembly of nanoEHS data for computational use (e.g., modeling and discovery), improved consistency for data interpretation, sharing, and interoperability, increased findability and longevity of data, support for sharing and transfer of knowledge between US federal partners and further scientific communities. To address the need for a consistent nomenclature for nanomaterials, here we discuss the creation of a consistent naming convention for EPA tested nanomaterials, with a focus on data included in the EPA NKB. We discuss our work with ISA-TAB-Nano and how this relates/improves input/output and data sharing. Further, we discuss semantic mapping and the NKB Resource Description Framework (RDF), as well as our creation of the EPA OntoSearcher tool that allows for automated ontology mapping. We illustrate here, through NKB use case examples, how this work contributes to a common language for nanoEHS data, which can be applied to other nanomaterials datasets, and is in support of FAIR data standards.

## Methods

### EPA-specific Nomenclature and Data Interoperability

**Figure 1.**
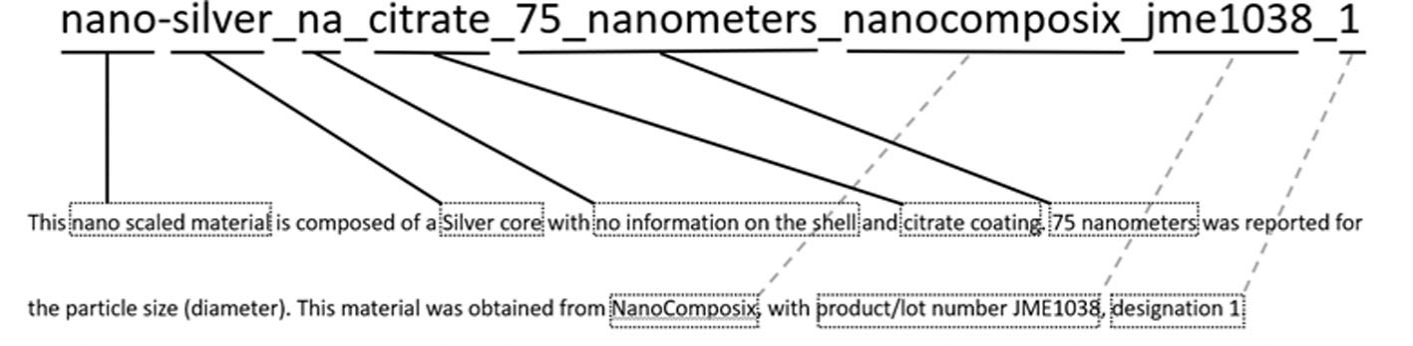
Unique names for materials in the NKB were generated using natural language processing (NLP) descriptions of the materials. These descriptions included physical, chemical, and commercial traits.

**Figure 2.**
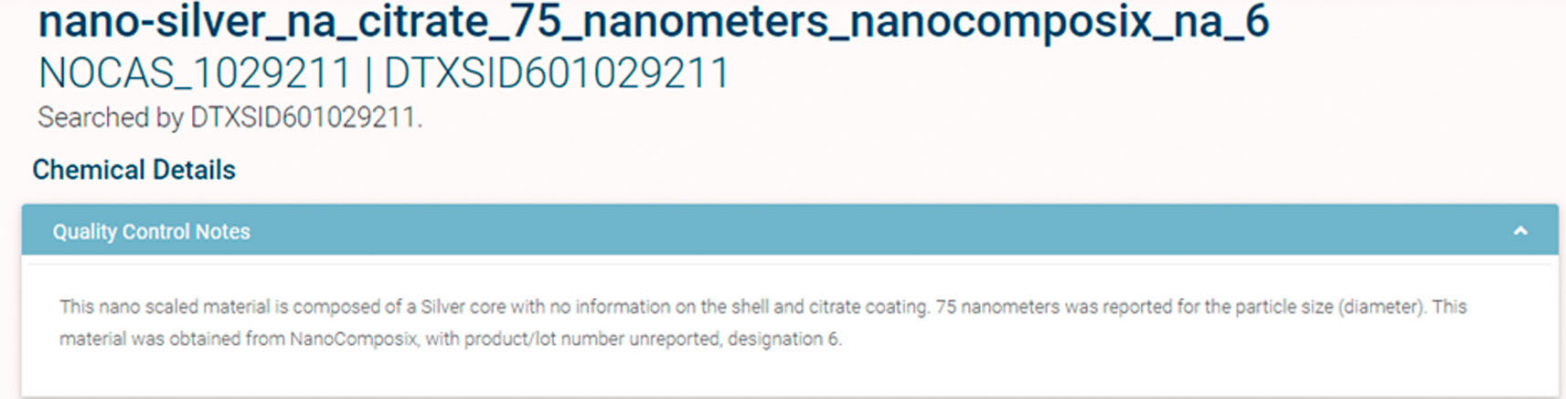
The NaKnowBase chemical substance list is accessible through the CompTox Chemicals Dashboard at https://comptox.epa.gov/dashboard/chemical-lists/naknowbase and provides access to each member of the list and their related substances.

**Figure 3.**
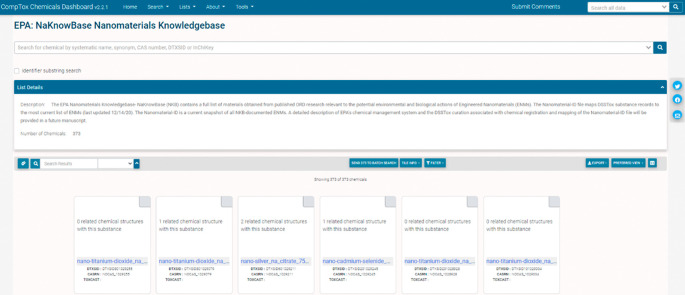
The name “nano-silver_na_citrate_75_nanometers_nanocomposix_na_6” maps onto the definition displayed in the Figure.

We have implemented an EPA-internal naming convention for nanomaterials using the Natural Language Processing (NLP) approach ( Figure 1), which incorporates chemical, physical, and mercantile elements of each material. A prefix of “nano” denotes that this is a nanomaterial, followed by the core material and any shell or coating information. Given the unique qualities of nanomaterials are often due to their size, we include the diameter. Any information on the supplier ID for the material (product or lot numbers) are subsequent, followed by an incrementing designation to track the number of separate materials with the said parameter in our dataset. Figure 2 illustrates how the substance name maps onto a definition of the makeup of the chemical. We have mapped all chemical substances in the NKB to the EPA CCD ( Figure 3).

We have developed Python code (version 3; ( Van Rossum 2009)) to automate formatting of ENM data into the standard and universally accepted ISO-TAB Nano format ( Marchese Robinson, Cronin et al. 2015). We have written this code to both WRITE (export) NKB data in ISO-TAB Nano format, entitled NKBtoITN.py, as well as READ (input) external data already in the ISO-TAB Nano format for potential inclusion into NKB, entitled ITNtoNKB.py.

**Figure 4.**
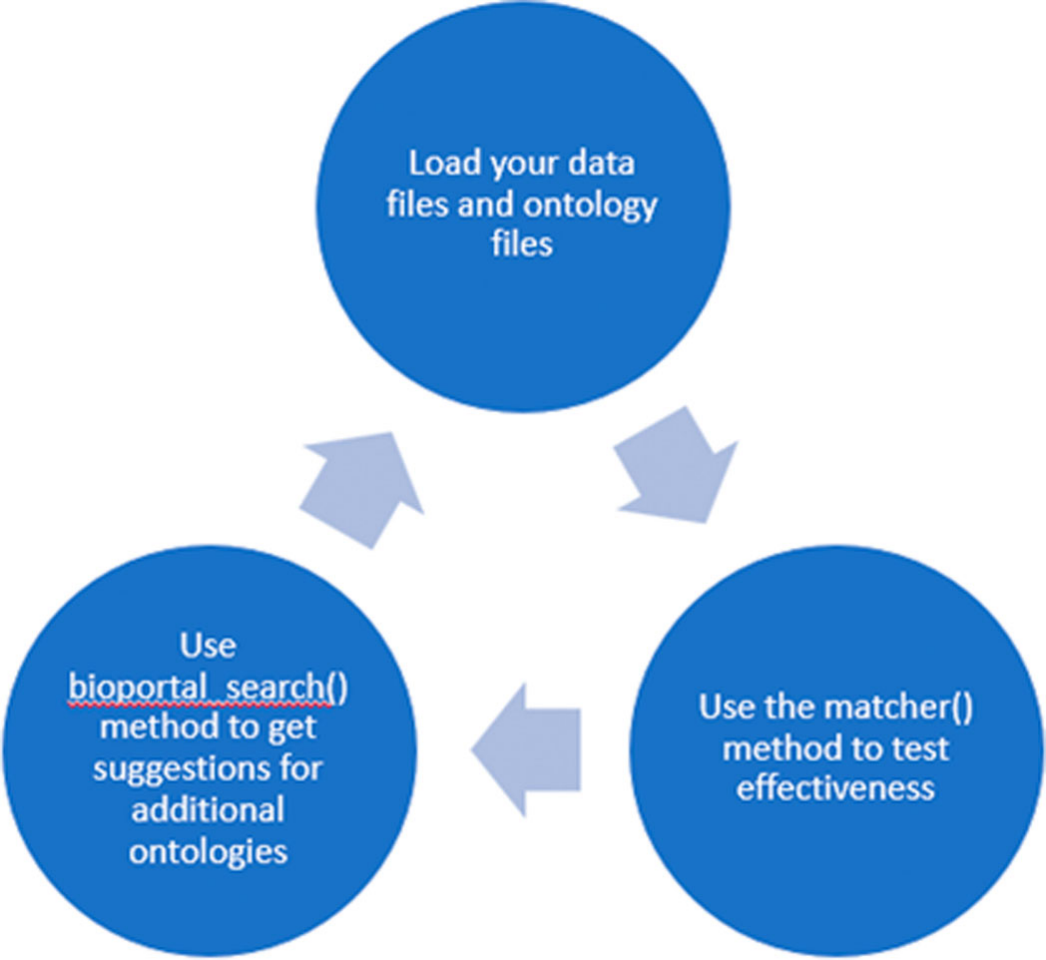
The first phase of the OntoSearcher workflow is an iterative exploration performed by the user and assisted by OntoSearcher. The user manually selects one or more ontologies, uses matcher() to see how well they cover the terms in the dataset, and is provided with suggestions for additional ontologies by bioportal_search(). This process continues until the user is satisfied with the coverage provided by the selected ontologies.

**Figure 5.**
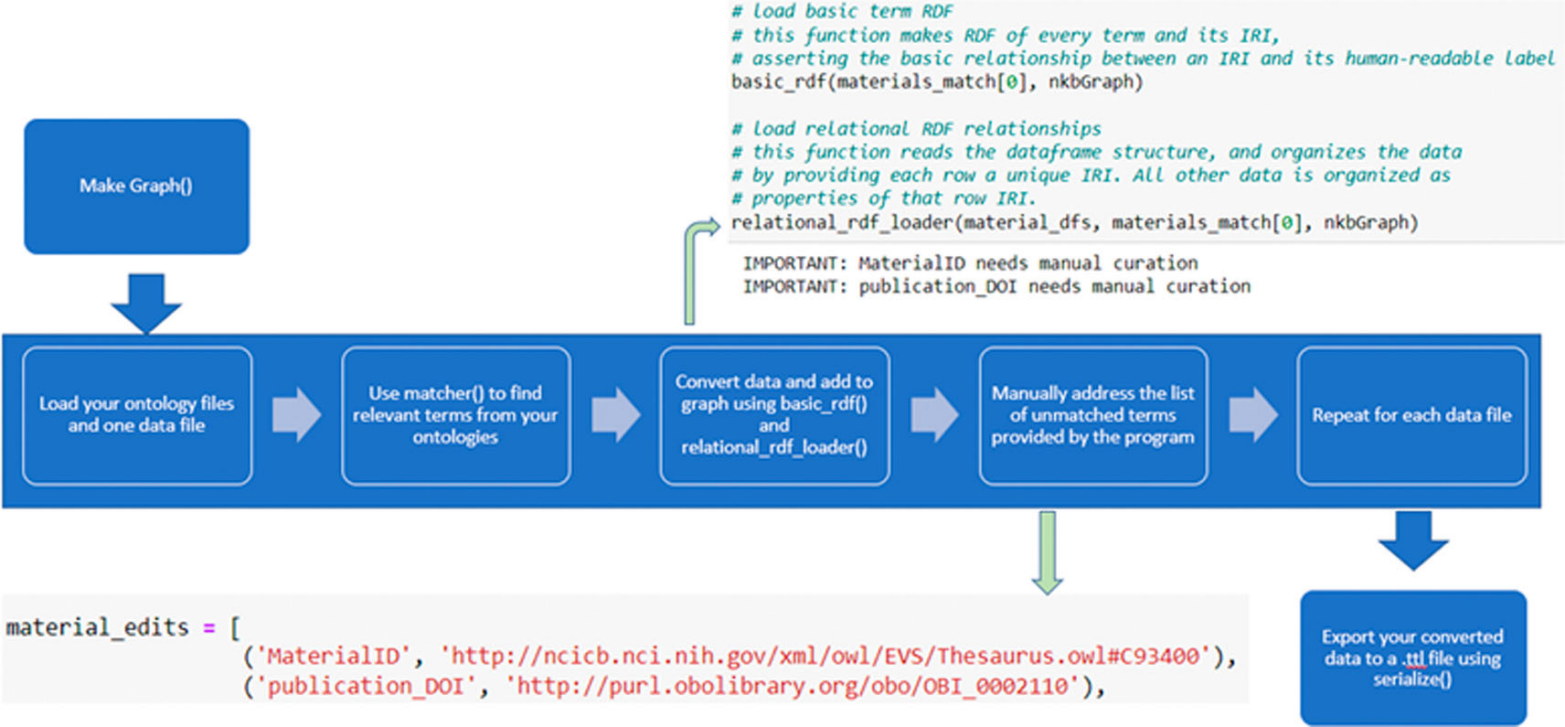
The second phase of the OntoSearcher workflow is a software-guided mapping of the dataset. The user processes each table from their dataset individually. The table is converted to triples, and the results of the matcher() method are used to automatically convert as many terms as possible into their equivalents from the ontologies. The remaining unmapped terms are reported to the user for manual curation. Once the manual mappings are supplied to Ontosearcher, it finishes handling the replacements.

**Figure 6.**
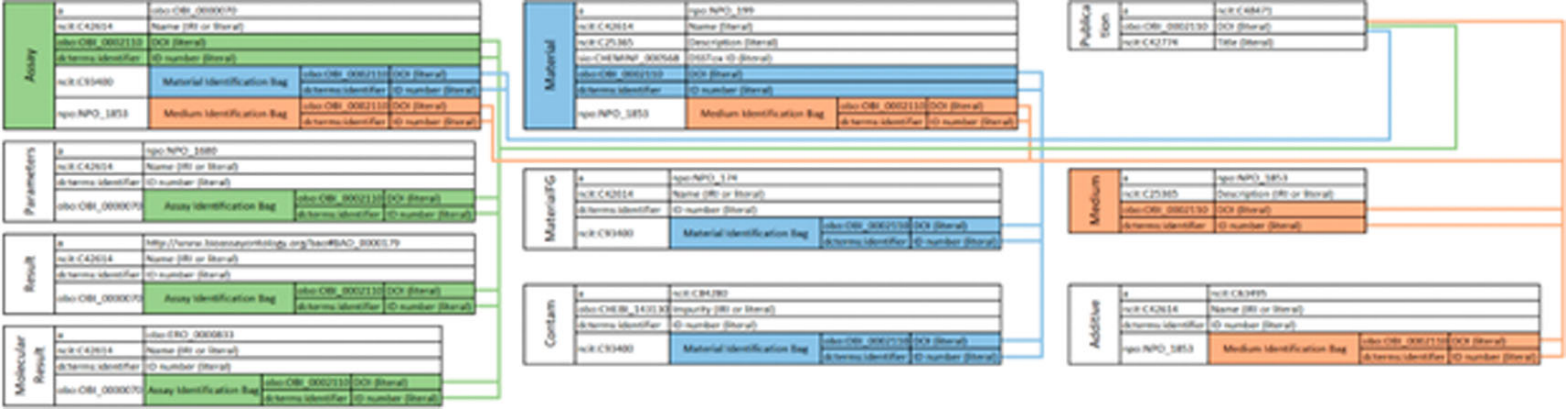
In each table, the first, vertically-alligned column lists the shared subject. The second column lists the predicate. The third column lists the object of the triple, which is either a simple object or a “bag” of triples sharing a common theme. Bags are further extended to show their predicates and objects. Connections are made through a combination of DOIs and IDs, represented by the lines in the schema. Since all data in the NKB is sourced from a paper, all types of data draw their DOI from the Publication triples. The IDs are used for differentiation of mediums, materials, and assays within a DOI. Mediums are marked in orange, materials are in blue, and assays are in green. This schema trims each table in the interest of space.

We have developed an application, entitled the EPA “OntoSearcher”. The OntoSearcher was developed in Python (version 3; ( Van Rossum 2009)) and is compatible with all major operating systems. Two types of input files are required to run the application: one or more .owl files (a standard format for ontology files, typically provided by an ontology owner when disseminated) and the user’s dataset in.csv format. If the user’s data were originally in a.sql format, one.csv file should be provided per table. Each data field should be a column, and each set of entries should be a row. Ontosearcher requires the packages ‘rdflib’ ( Boettiger 2018) to create and manage the graph, ‘rapidfuzz’ ( Bachmann 2021) to handle fuzzy matching during searches, and ‘owlready2’ ( Lamy 2017) to assign ontologies. OntoSearcher workflows for term matching and graph creation are provided in Figure 4 and Figure 5, respectively. Figures 6 illustrates the data tables resulting from the semantic mapping of NKB.

Interactive tutorials ( **S1, S2**) have been developed using the Jupyter Notebook document format ( Thomas Kluyver 2016). Use of OntoSearcher requires an Application Programming Interface (API) key to grant software access to a service and for the BioPortal web service ( Whetzel, Noy et al. 2011). Input files are required in the.csv and.owl formats. An intermediate file is created in.json format. Output files are written in terse RDF triple language format or.ttl (( W3C team 2011); http://www.w3.org/TeamSubmission/turtle/), which is a text file format. The resulting.ttl file can be used directly by parties of interest, or alternatively, used to establish a SPARQL endpoint (( W3C team 2013); https://www.w3.org/TR/sparql11-overview/) for distribution and sharing of the RDF. Table 1 lists the publicly available ontologies and vocabularies used the in semantic mapping of NKB, each sources prefix in RDF and associated IRIs.

**Table 1.** Overview of ontologies, vocabularies, and other data sources present in the NKB RDF.

Name	Prefix in RDF	IRI
**Biomedical Informatics Research Network Project Lexicon**	**birnlex**	http://bioontology.org/projects/ontologies/birnlex#
**Dublin Core Metadata Element Set**	**dc**	http://purl.org/dc/elements/1.1/
**Dublin Core Terms**	**dcterms**	http://purl.org/dc/terms/
**EDAM Ontology Ison et al. (2013)**	**edam**	http://edamontology.org/
**eNanoMapper Ontology**	**enm**	http://purl.enanomapper.org/onto/
**National Cancer Institute Thesaurus**	**ncit**	http://ncicb.nci.nih.gov/xml/owl/EVS/Thesaurus.owl#
**NanoParticle Ontology**	**npo**	http://purl.bioontology.org/ontology/npo#
**Berkeley Bioinformatics Open-source Projects Ontologies**	**obo**	http://purl.obolibrary.org/obo/
**The Gene Ontology**	**oboInOwl**	http://www.geneontology.org/formats/oboInOwl#
**DataONE Ontologies**	**odo**	http://purl.dataone.org/odo/
**RDF Concepts Vocabulary**	**rdf**	http://www.w3.org/1999/02/22-rdf-syntax-ns#
**RDF Schema Vocabulary**	**rdfs**	http://www.w3.org/2000/01/rdf-schema#
**Semantic Science Integrated Ontology**	**sio**	http://semanticscience.org/resource/

## Results

The NKB schema ( Boyes, Beach et al. 2022) was designed to capture a material, the parameters of experiments performed upon it, and the results of those experiments. In order to improve the interoperability of NKB and other datasets, we implemented an EPA-internal naming convention for nanomaterials, depicted in Figure 1. This naming scheme was established using the Natural Language Processing (NLP) approach, which incorporates chemical, physical, and mercantile elements of each material. The utility of this approach is illustrated in Figure 2, whereby name of the substance maps onto a definition of the makeup of the chemical. While this naming convention is useful for EPA internal cases, such as connecting with the EPA CCD, it is not ideal for expanded comparisons because it may not contain enough information to remain distinguishable against other datasets (e.g., data that may not have particular parameters contained within NKB or CCD). Currently, we have 373 chemical structures mapped on the EPA CCD, available at https://comptox.epa.gov/dashboard/chemical_lists/NAKNOWBASE, and illustrated in Figure 3. This collaborative, cross EPA-ORD effort is updated as NKB ENMs are made available and added for curation.

We have implemented the spreadsheet-based format for nanomaterials sharing, ISA-Tab-Nano, which was inspired by the high-throughput genomics community ( Thomas, Gaheen et al. 2013). Both pieces of code WRITE/OUTPUT (NKBtoITN.py), and READ/INPUT (ITNtoNKB.py) and corresponding documentation are made available to the public via EPA ORD and Data.gov and available for download ( CSS_3.2.2.1_NKB_IsoTabNano.zip) at https://catalog.data.gov/dataset/css-3-2-2-1-naknowbase. Though this code is useful for specifically NKB to ISA-Tab-Nano conversion, we found that extending this code to other datasets was problematic; whereby, other data would have to be re-formatted to NKB format or code would need to be rewritten to accept alternate input formats.

To continue to improve NKB interoperability, we have developed an application, entitled “OntoSearcher”, that automates ontological term mapping for a given ENM dataset. We have developed this python code to read in ENM data from NKB, and generically for other data sources, map those data to ontological terms, and provide a corresponding diagnostic report on speed and accuracy of the mapping. The code and corresponding documentation for the tool and application, as well as training materials, are made available to the public via EPA ORD and Data.gov and available for download (OntoSearcher_Training_Materials.zip) at https://catalog.data.gov/dataset/naknowbase-interoperability-tools. Schematics of two OntoSearcher workflows, Phase 1 and Phase 2 are presented in Figure 4 and Figure 5, respectively. As illustrated in Figure 4, the Phase 1 OntoSearcher workflow illustrates the conversion of an existing dataset into an RDF. This process required two types of input files (i.e., .owl and.csv). The OntoSearcher ‘ *matcher*’ method evaluates how well the supplied ontologies cover the dataset. In order to provide additional coverage of the dataset, ontologies are identified by bioportal search ( Noy, Shah et al. 2009) in a ‘ *search*’. The user iterates between ‘ *matcher*’ and ‘ *search*’ until their collection of ontologies covers a strong majority of their dataset. Lastly, when percentage of matches is achieved, the combined ontologies are output to file. In the second phase, shown in Figure 5, the dataset is added in table increments, to a graph. The ‘ *matcher*’ method is used again, this time to determine which terms have been matched by the finalized ontologies and identify those terms that require manual curation. Terms are returned as either a matched group or an unmatched group, allowing the curator to review the automated matches. Term replacements are performed on the graph, whereby the natural language terms are swapped out from the raw data with the matching terms from the compiled ontologies. Next, the curator consults their ontologies to find matches for the unmatched terms. When the list of manual curations is complete, it is applied to the graph. In some cases, implied relationships between columns in the dataset will need to be explicitly stated by the curator by using the graph search method, such as a ‘nodebag’ method (e.g., ‘ *diameter value’* and ‘ *diameter units’* columns are both related to the concept of “diameter” to a human reader, but such relationships are not observed by the computer). Once the data have been matched to and replaced by the correct ontology terms, they are ready to be serialized into a.ttl file.

Figure 6 illustrates the NKB RDF schema produced using the OntoSearcher tool and following the above procedures for semantic mapping of ten NKB tables that correspond to Boyes, et al. ( Boyes, Beach et al. 2022) (i.e., Assay; Parameters; Result; Molecular Result; Material; Material FG; Contam; Publication; Medium; Additive). The NKB RDF has 10,261,162 triples, documenting 22,329 assays performed on a combined 373 materials from 128 publications. Table 1 lists each of the established and publicly available ontologies and vocabularies used in mapping, manual and automated, to terms in NKB tables illustrated in Figure 6.

**Figure 7.**
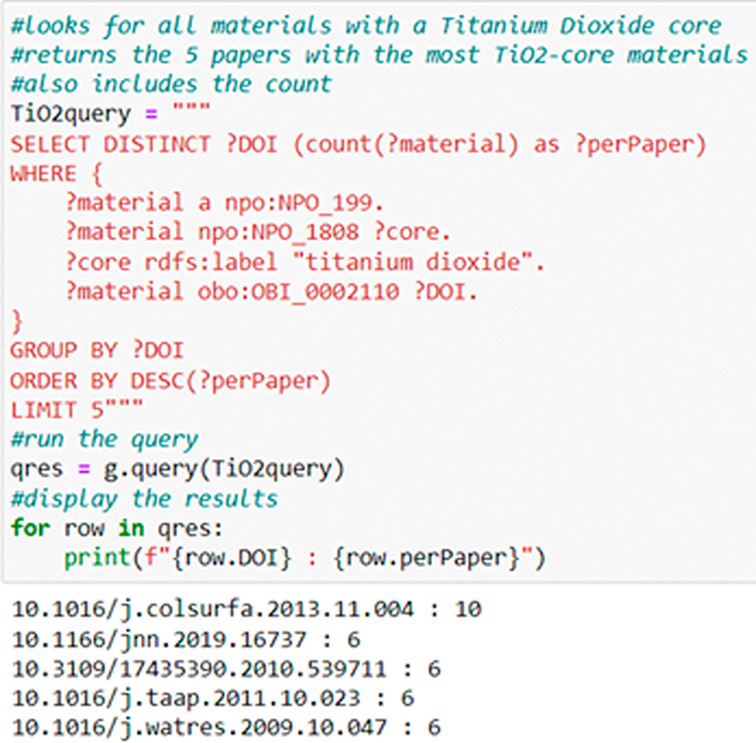
A simple query on the NaKnowBase RDF. This query finds all materials in the NaKnowBase listed as having a Titanium Dioxide core, then groups the results by source publication and limits the display to the top 5.

**Figure 8.**
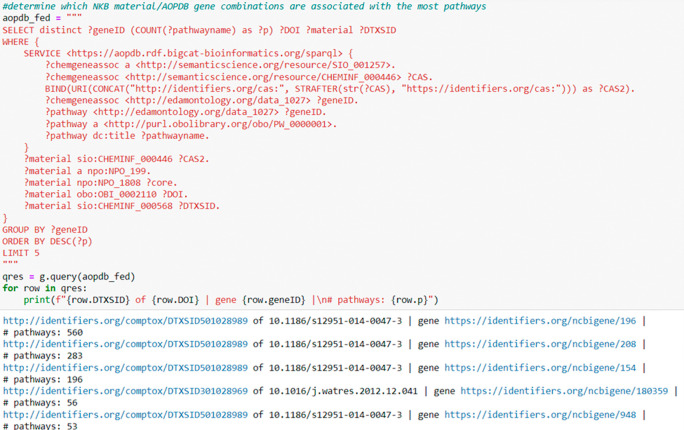
A federated query using the NaKnowBase RDF and the AOPDB SPARQL endpoint. This query calls to the AOPDB SPARQL endpoint for information on the relationships between materials (by CASRN), genes, and pathways. Then, locally, the results are used to query the NaKnowBase RDF and determine which NKB materials correspond to the results from the AOPDB. The results show the 5 material-gene combinations that impact the most pathways, as well as the DOI of the source paper for that material in the NKB. The materials are reported by link to the CompTox Chemicals Dashboard.

Figure 7 Illustrates a local (NKB) SPARQL use case query for publications included in NKB that contain data on ‘Titanium Dioxide core’, specifically. Results of this query are requested by publication DOI, and limited to 5 for illustration purposes. Figure 8 illustrates a federated SPARQL query across two EPA datasets, NKB ( Boyes, Beach et al. 2022) and AOP-DB ( Mortensen, Senn et al. 2021), where the user is inquiring as to which NKB nanomaterial is associated with the highest number of biological pathways in AOP-DB. Results are again limited to 5 for ease of illustration.

## Discussion

In an effort to improve the EPA NKB from the relational database presented by Boyes et al. (2022), we have developed tools and applications which promote the development of a common language for ENMs and contribute to “FAIRification” of US Federal ENM data. The purpose of these tools are to enable improved assembly of nanoEHS data for computational use with minimized human curation, improved consistency of data format for interoperability, increased findability and longevity of nanoEHS data, and to enable support for sharing and transfer of knowledge between US Federal partners and further scientific communities.

Because manual curation for even a small dataset is not trivial, we created EPA OntoSearcher to semi-automate the process of mapping pre-defined ontological terms to individual datasets, and thereby minimizing manual input from the curator. We have developed code to read in ENM data from NKB and other data sources, and map those data to ontological terms, as well as providing a corresponding diagnostic report on speed and accuracy of the mapping. We walk the user through this process, as illustrated in the OntoSearcher workflows presented in Figures 4 and 5. We present our own use of the tool, with the EPA NKB semantic mapping illustrated in Figure 6, using the ontologies listed in Table 1. Multiple queries were performed on the NKB RDF using the locally stored.ttl file. Figure 7 contains a simple example, in which the.ttl file is queried for information on every instance of Titanium Dioxide in the dataset. Figure 8 contains a more advanced, federated query example, showing the benefits of the RDF format in traversing across datasets. Since the AOP-DB RDF is available through a SPARQL endpoint ( Mortensen, Martens et al. 2022), we were able to perform a query directly on both datasets, listing any materials from the NKB that are involved in AOPs derived from the AOP-Wiki, and include information on which genes are relevant to the interaction.

With the RDF, formatting is reduced to an object, a relationship, and the target of that relationship (i.e., the “ *triple*”). All data, however complex, can be reduced to some number of sets of these triples. The terms used as each part of a triple are drawn from rigidly-defined ontologies. Many ontologies already exist to provide structure to scientific vocabularies. Some of these ontologies, such as the Chemical Information Ontology (cheminf) (( Fu 2021); https://www.ebi.ac.uk/ols/ontologies/cheminf), are broad enough to be useful for reducing some aspects of nanomaterial data to triples ( Hastings, Jeliazkova et al. 2015). However, unique disciplines require additional support for total coverage.

With the increase in study of nanomaterials over the last decades, we have seen an increase in nano-related databases, knowledgebases, web-based libraries, and registry repositories ( Ayadi, Rose et al. 2021) for review). Further, the importance of nanomaterial-specific ontologies in relation to data-sharing has been recognized in many nano and nano-related subject areas ( Thomas, Klaessig et al. 2011, Hastings, Jeliazkova et al. 2015, Jeliazkova, Chomenidis et al. 2015, Karcher, Willighagen et al. 2018, Ayadi 2020, Ayadi, Rose et al. 2021). Given the vast breadth of information, and format across these datasets, it becomes impractical and even untenable to carry out any data integration exercise that would allow a concerted observation, such as that necessary for regulatory decision-making. This observation prompts a dramatic need for a shift in thinking from recent nanoinformatics initiatives and efforts at nanomaterial data curation for specific purposes ( National Science and Technology Council 2011, Hendren, Powers et al. 2015, Karcher, Willighagen et al. 2018, Yan, Sedykh et al. 2020). Further, given the current speed at which nanomaterials enter the marketplace, collecting and curating data on emerging nanomaterials is expected to become more difficult over time. Both the number of nanomaterials and the scope of characteristics to catalog are constantly increasing. Tools that assist and automate aspects of data processing, like EPA OntoSearcher, will become increasingly useful in our ability to maintain consistent curation standards, interpret, analyze and share data, while reducing risk of computer-introduced errors or biases. For example, automated tools can compare a term against thousands of definitions and synonym lists in the time it takes the human curator to compare against a single definition. Proper use of these tools reduces the curator’s workload to merely validating the matches made by the tool and handling whatever terms were absent from ontologies. Rapid integration of disparate data sources, enabled by these sorts of toolkits, will greatly affect nanoinformatics on all levels from the data customer, creator, and curator, to the analyst or assessor. Our ability to scrutinize multiple data sources consecutively allows for more efficient and timely understanding of emerging nanomaterials, while underlining the central role of data standardization, interoperability and FAIR practices in our ability to meet the incoming needs for interpretation of the effects of nanomaterials on the environment and public health.

## Conclusions

The methods, tools and applications described here will be implemented for the inclusion, processing and FAIRification of novel EPA data related to nanomaterials. EPA and other Federal partners, as part of the NEHI DIIG, have proposed a federal nanoEHS consortium to create interoperable formats for federal nanoEHS data. The results of this effort are forthcoming.

## Author contributions

HM: Conceived of, directed the study and analyses, and wrote and edited the manuscript; BB: revised the NKB RDF to version 2, improved the OntoSearcher tool, developed training documents, produced figures, wrote and edited the manuscript; WS: developed code for the initial version of OntoSearcher; JS: provided technical support and expertise for the first version of OntoSearcher and NKB RDF version 1; AW: Performed the NLP and mapping of NKB materials to DSSTox, assisted in creating the EPA nomenclature for NKB, created the NKB nanomaterial linkages in CCD and wrote and edited the manuscript; WB: wrote and edited the manuscript.

## EPA disclaimer

This manuscript has been reviewed by the Center for Public Health and Environmental Assessment, United States Environmental Protection Agency and approved for publication. Approval does not signify that the contents necessarily reflect the views and policies of the Agency nor does mention of trade names or commercial products constitute endorsement or recommendation for use. The authors declare no conflict of interest.

## ORISE disclaimer

This research was supported in part by an appointment to the U.S. Environmental Protection Agency (EPA) Research Participation Program administered by the Oak Ridge Institute for Science and Education (ORISE) through an interagency agreement between the U.S. Department of Energy (DOE) and the U.S. Environmental Protection Agency. ORISE is managed by ORAU under DOE contract number DE-SC0014664. All opinions expressed in this paper are the author’s and do not necessarily reflect the policies and views of US EPA, DOE, or ORAU/ORISE.

## Data Availability

The U.S. EPA has made NaKnowBase data files, and all related code and training materials discussed here, publicly available through the EPA Office of Research’s Science Hub at: DOI: http://10.0.92.167/1523156, which feeds to Data.gov at https://catalog.data.gov/dataset/naknowbase-interoperability-tools. Source data for the entire NaKnowBase relational database ( Boyes, Beach et al. 2022) is available at: https://catalog.data.gov/dataset/css-3-2-2-1-naknowbase, and at: https://gaftp.epa.gov/EPADataCommons/ORD/NaKnowBase/.
