# Supplementary material for: Translating nanoEHS data using EPA NaKnowBase and the resource description framework
Source: F1000Res. 2024 Mar 8;13:169. [Version 1] doi: 10.12688/f1000research.141056.1 (PMC11128042; doi:10.12688/f1000research.141056.1)
Supplement: Peer review history

# Reviewer report: Reviewer response for version 1

**DOI:** 10.5256/f1000research.154467.r269340

**Published:** 2024-05-25

**Authors:** Philip Doganis

**Affiliations:** 1 Unit of Process Control and Informatics, School of Chemical Engineering, National Technical University of Athens, Athens, Greece

This work presents methods and tools to include, process and FAIRify NM data, whether new or not published in a machine-readable format with sufficient accompanying information. It provides an efficient and easy to use workflow that can lead to FAIR datasets. As with all automatic tools, the semantic integration OntoSearcher tool has some limitations inherent in the rationale structured into it, but if a tool can handle the straightforward terms and leave some ontology terms for the researcher that is great help in an otherwise daunting task. Perhaps an explanation of the parameterisation of the tool would be needed, so more experienced users can intervene. 

 Overall, this is needed work that solves an existing problem.

 Change ISOTAB to ISATAB in linked documents, (i.e. in https://catalog.data. gov/dataset/css-3-2-2-1-naknowbase )

 Brief mention of background and need for this and its interactions with existing work.

 interoperability with U.S. and European partners ?

 US-EU roadmap, is there a citation?

 p.4 “Efforts to coordinate data have been hindered by diversity of information, and format incongruities. “ Also mention missing or partial description of protocols

 since modelling is mentioned, are there scripts that enable easy extraction in a ready-to-model format?

 p5. Define the connection to, or possible assignment of, DTXSIDs for the NMs. Will they be usable in the EPA tools (i.e. CCTE ?)

 Fig.6 provide in higher resolution or provide an external link where it is legible

 p.8, 10 How does Ontosearcher handle multiple ontology results for a term? (does it choose the first?)

 p.8 The phrase “(e.g., ‘diameter value’ and ‘diameter units’ columns are both related to the concept of “diameter” to a human reader, but such relationships are not observed by the computer)” should be rephrased for clarity. By refering “are not observed by the computer” does this refer to the processing of the data?

 For example, there this entry:

https://bioportal.bioontology.org/ontologies/OM?p=classes&conceptid=http%3A%2F%2Fwww.ontology-of-units-of-measure.org%2Fresource%2Fom-2%2FDiameter

 which is an example of the possibility to refer to diameter units of measure.

Are sufficient details of methods and materials provided to allow replication by others?

Yes

Is the rationale for creating the dataset(s) clearly described?

Yes

Are the datasets clearly presented in a useable and accessible format?

Yes

Are the protocols appropriate and is the work technically sound?

Yes

Reviewer Expertise:

NA

I confirm that I have read this submission and believe that I have an appropriate level of expertise to confirm that it is of an acceptable scientific standard.

# Reviewer report: Reviewer response for version 1

**DOI:** 10.5256/f1000research.154467.r263181

**Published:** 2024-05-08

**Authors:** Vladimir Lobaskin

**Affiliations:** 1 University College Dublin, Dublin, Leinster, Ireland

The manuscript describes new tools facilitating the annotation and integration of nanomaterials data from EPA NaKnowBase. The motivation part outlines the current challenges in the NanoEHS area and proposes a solution following the FAIR principles and direction from the EU-US Nanoinformatics Roadmap. The developed methodology and software have obvious utility and are indispensable for current and future nanosafety research. The methodology is well justified and illustrated, and, most importantly, linked to the web resources. I am happy to recommend this paper with the following minor amendments.  

 It would be good to mention the paper on the latest major EU-US effort (Maier D, et al., 2023 [Ref 1])

 Typos:

 Page 3: “Nanoinformatics, the compilation, integration, and assessment of nanoEHS data”  - Generally, nanoinformatics entails more than this. It also includes data processing and management, annotation, ontologies, modelling tools, etc. I suggest rephrasing it as follows: “Nanoinformatics, the field concerning in particular the compilation, integration, and assessment of nanoEHS data” or add a more complete definition. 

 Page 4: “agency specific” ->  agency-specific

 Page 4: “and, to our knowledge is” -> and, to our knowledge, is

 Page 4,5: “ISO-TAB Nano” (3 instances)  > ISA-TAB-Nano

 Page 5: “in.csv format. If the user’s data were originally in a.sql format, one.csv

 file should be provided per table.”  - > in .csv format. If the user’s data were originally in a .sql format, one .csv file should be provided per table.

 Page 5: “Ontosearcher” -> OntoSearcher

 Page 5: “Figures 6 illustrates” -> Figure 6 illustrates

 Page 5: “the.csv and.owl” -> the .csv and .owl

 Page 5: “ in.json”, “resulting.ttl”, “or.ttl”  ->  in .json, resulting .ttl, or .ttl

 Page 6, Figures 4,5: redraw to remove the underlining of “bioportal_search()”, “.ttl”

 Page 6: “Natural Language Processing (NLP) approach (Figure 1), which incorporates chemical, physical, and mercantile elements of each material.”  - repetition of a sentence from the Methods section.

 Page 7:  “ISA-Tab-Nano” ->  ISA-TAB-Nano.

 Page 8:  “and.csv” -> and .csv

 Page 8:  “when percentage of matches is achieved” – desired percentage or final percentage? It is better to be specific.

 Page 8: “The purpose of these tools are” -> The purpose of these tools is

 Page 8: “stored.ttl”, “the.ttl” - > stored .ttl, the .ttl

 Page 10: “registry repositories (Ayadi, Rose et al. 2021) for review).” -> registry repositories (see (Ayadi, Rose et al. 2021) for review).

 Page 11: “Haase AKF e” -> Haase A, Klaessig F

Are sufficient details of methods and materials provided to allow replication by others?

Yes

Is the rationale for creating the dataset(s) clearly described?

Yes

Are the datasets clearly presented in a useable and accessible format?

Yes

Are the protocols appropriate and is the work technically sound?

Yes

Reviewer Expertise:

Nanoinformatics, Materials science, Condensed Matter Physics

I confirm that I have read this submission and believe that I have an appropriate level of expertise to confirm that it is of an acceptable scientific standard.

## References

[1] : Harmonising knowledge for safer materials via the “NanoCommons” Knowledge Base. *Frontiers in Physics* .2023;11: 10.3389/fphy.2023.1271842 10.3389/fphy.2023.1271842
